# Immunoactivity of self-assembled antibodies investigated by atomic force microscopy†

**DOI:** 10.1039/c8ra05423a

**Published:** 2018-08-20

**Authors:** Hiroaki Kominami, Kei Kobayashi, Shinichiro Ido, Hirokazu Kimiya, Hirofumi Yamada

**Affiliations:** Department of Electronic Science and Engineering, Kyoto University Katsura, Nishikyo Kyoto 615-8510 Japan yamada@kuee.kyoto-u.ac.jp; Advanced Research Division Device Research Laboratory, Panasonic Corporation 3-1-1 Yagumo-naka-machi Moriguchi City Osaka 570-8501 Japan

## Abstract

Immunoglobulin G (IgG), an antibody, plays a significant role in the immune system, and the functions of IgG molecules have been studied in many research fields such as medicine and engineering. Recently, we found the self-assembly of monoclonal mouse IgG molecules on a mica substrate using atomic force microscopy (AFM); the IgG molecules self-assemble into hexamers and the hexamers form a two-dimensional (2D) crystal. The self-assembly of the IgG molecules is of great interest in terms of the enhancement of the immunoactivity of the antibodies. In this study, we investigated the self-assembly of various IgG molecules on a mica substrate to discuss if the hexamerization of the IgG molecules is a general phenomenon. We also investigated the antigen binding site in the IgG antibody hexamers, and estimated the association rate constant of the self-assembled IgG molecules based on the AFM measurements. The estimated value was lower than that reported in a previous study probably because of the limited mobility of the antigen-binding fragments on the substrate.

## Abstract 2

We investigated self-assembly such as hexamerization and two-dimensional crystallization of immunoglobulin G (IgG) molecules on mica by atomic force microscopy. We also estimated the association rate constant of the self-assembled IgG antibodies.

## Graphical abstract

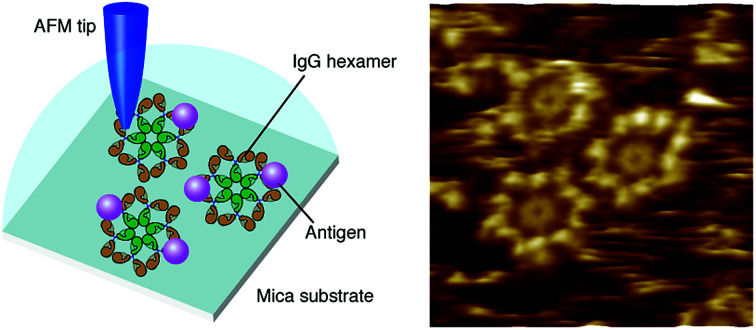

## Introduction

**Fig. 1:**
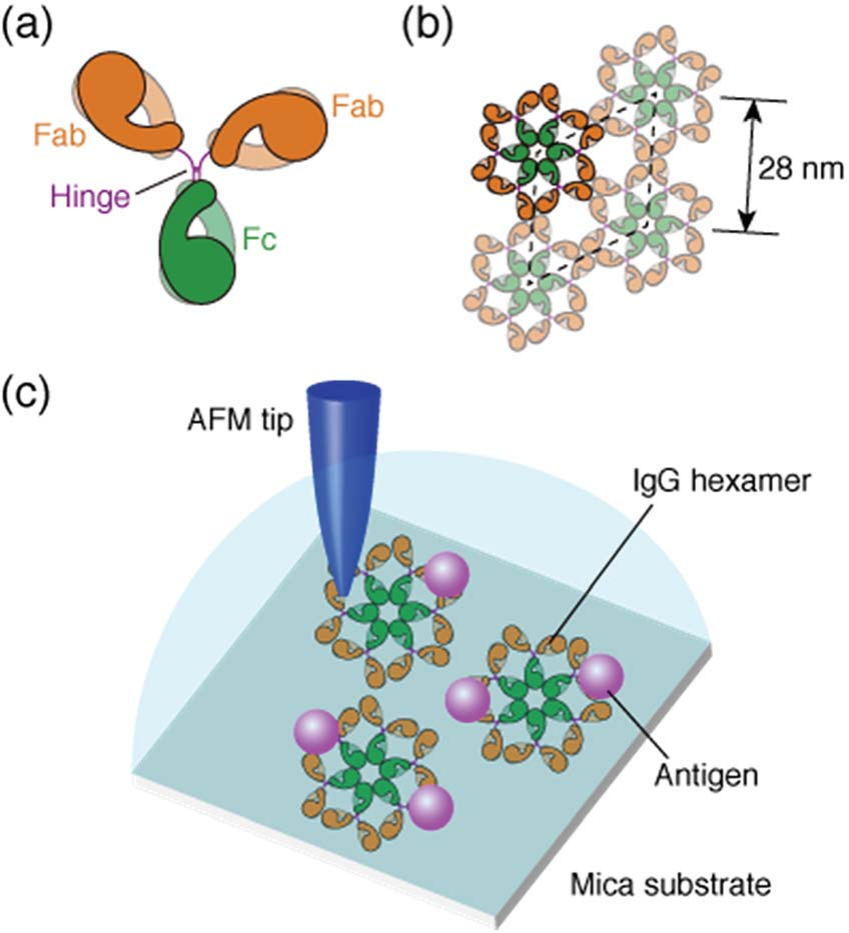
Structural model of IgG antibodies. (a) Isolated IgG antibody molecule and (b) hexameric IgG antibodies in 2D crystal. (c) Schematic of experimental protocol; AFM imaging of the self-assembled IgG hexamers and the interaction of the antigenic molecules on the 2D IgG crystals.

The antibody, also known as an immunoglobulin (Ig), is a protein that plays essentially important roles in the immune system by binding to its specific antigen. The antibodies are composed of two heavy chains and two light chains. They are categorized into classes, such as IgG, IgM, IgA, IgD and IgE, depending on the type of the heavy chain, and further categorized into subclasses by the amino acid sequences of the heavy chain.^1^ IgG, the most abundant antibody in human serum has been studied in many research fields such as medicine and engineering.^2^ The IgG antibodies have a Y-shaped structure that consists of two antigen-binding fragments (Fab region), one crystallizable fragment (Fc region), and the hinge region^3,4^ (Fig. 1a). The heavy chains constitute the Fc region and a part of the Fab region, and the light chains constitute the rest of the Fab region. The heavy and light chains are connected with each other by disulfide bonds. The amino acid sequences in the Fc region and a part of the Fab region are very similar in the IgG antibodies of a species (constant region), but are different among the species. The remaining part of the Fab arm is the variable region, a part of which is the complementarity determining region (CDR) that binds to a unique epitope in the antigen.^5^

The immune functions, such as phagocytosis, are mediated by the complement proteins. Following the binding of the IgG or IgM antibody to the antigen, the antibody binds to C1q, a subcomponent of the complement protein C1. This triggers the complement cascade that eventually leads to the phagocytosis. It has been reported that the affinity between antibodies and C1q is enhanced by self-assembly of the antibodies.^6–8^ While the IgM molecules exist in a pentameric form, the IgG molecules exist in a monomeric form, but it was recently shown that the IgG molecules form a hexamer and the IgG hexamer binds to the complement complex by transmission electron microscopy.^9–11^ Recently, we also reported the self-assembly of monoclonal IgG antibodies from mouse^12^ using high-resolution atomic force microscopy (AFM)^13–20^ (Fig. 1b). We found that they form hexamers on a mica substrate. Although the IgG1 antibodies from mouse are not capable of activating the complement,^21^ we believe that the hexamer formation is related to the function of the IgG antibodies.

In a previous study, we also reported the two-dimensional (2D) crystal formation of the antibody hexamers on the mica substrate. We have shown the adsorption of specific antigenic molecules on the 2D IgG crystals. Since the antibody hexamers are uniformly distributed in the 2D crystal on the substrate, it is a good platform to study the immunoactivity of the self-assembled antibodies by AFM.

In this study, we investigated the generality of the hexamer formation for various IgG antibodies using AFM. We also investigated the antigen binding site in the IgG antibody hexamers using AFM. Furthermore, the association rate constant of the IgG antibodies in the antibody 2D crystal was estimated by the AFM measurement. A schematic of the experimental protocol is presented in Fig. 1c.

We used anti-human serum albumin (anti-HSA) monoclonal IgG antibodies from mouse that we used in a previous study,^12^ referred to as “IgG1-mouse-A” hereafter, and four other IgG antibodies. They are the same IgG1 antibody from mouse, but with a CDR different from the IgG1-mouse-A, referred to as “IgG1-mouse-B”, the antibody from the mouse but of a different subclass IgG2a (“IgG2a-mouse”), polyclonal antibodies from the mouse that include several subclasses of the IgG antibodies (“IgG-mouse”), and the IgG1 antibody from a rat (“IgG1-rat”).

## Experimental

### Antibody solution

Two kinds of anti-HSA mouse monoclonal antibodies (IgG1 isotype; “IgG1-mouse-A” and “IgG1-mouse-B”) were isolated from ascites fluid of a mouse and purified by using protein A and dialysis with phosphate-buffered saline. The antibodies were dissolved into 10 mM phosphate buffer solution (pH 7.5, Sigma-Aldrich) to obtain a solution with a concentration of 2.0 μM (IgG1-mouse-A) or 0.2 μM (IgG1-mouse-B). We purchased anti-actin mouse monoclonal antibodies (IgG2a isotype, “IgG2a-mouse”) from Sigma-Aldrich, anti-monocarboxylic acid transporter 1 mouse polyclonal IgG antibodies (“IgG-mouse”) from Abcam, and anti-mouse tumor necrosis factor-α rat monoclonal antibodies (IgG1 isotype, “IgG1-rat”) from R&D Systems. The antibodies were dissolved into 10 mM phosphate buffer solution to obtain a solution with a concentration of 1.0 μM (IgG2a-mouse), 0.68 μM (IgG-mouse) and 0.34 μM (IgG1-rat). A 5 μl droplet of the antibody solution was dropped onto a fleshly cleaved mica substrate (10 × 10 mm^2^, Furuuchi Chemical) with a 5 μl droplet of 10 mM phosphate buffer solution (pH 7.5) containing 50 mM magnesium chloride hexahydrate (MgCl_2_·6H_2_O, 99.999% purity, Alfa Aesar) (“imaging solution”).

### Antigenic molecule

Human serum albumin (HSA) purchased from Sigma-Aldrich was used as an antigenic molecule. HSA was dissolved in 10 mM phosphate buffer solution (pH 7.5).

### AFM imaging

We used a lab-modified AFM instrument (SPM-9600, Shimadzu) and a lab-built controller programmed in LabVIEW (National Instruments). Low thermal drift was achieved by placing the AFM instrument in a temperature regulated enclosure (CN-40A, Mitsubishi Electronic Engineering). We performed AFM imaging in the constant frequency shift mode of frequency-modulation AFM in the forementioned “imaging solution” at 24 °C. We used silicon cantilevers (PPP-NCHAuD, Nanosensors) with a Au backside coating. Nominal spring constant was about 42 N m^−1^ and resonance frequency in liquids was about 130 kHz. A nominal tip radius was 7 nm. The displacement noise density was reduced approximately less than 20 fm Hz^−1/2^. Typical oscillation amplitude was set at 0.5 nm. WSxM^22^ (Nanotech Electronica) was used to analyze AFM images.

## Results and discussion

**Fig. 2:**
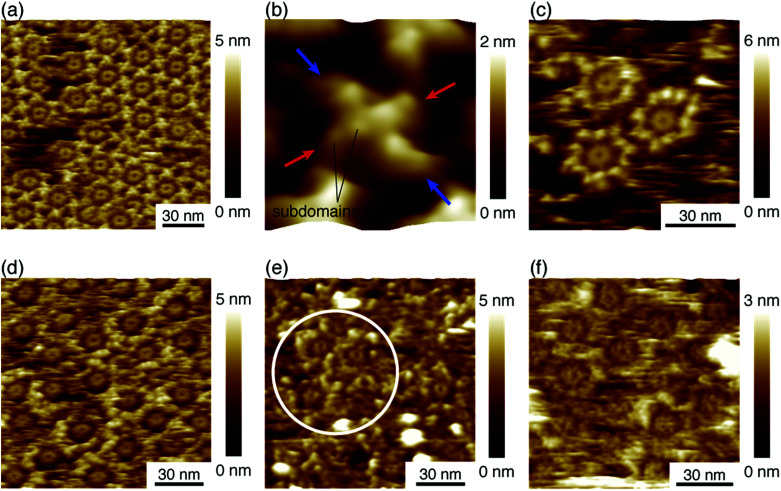
AFM images of hexameric IgG antibodies. (a) 2D IgG crystal of hexameric IgG1-mouse-A. (b) High-resolution image of X-shaped structure consisting of four Fab regions. (c) Hexameric IgG1-mouse-B antibodies, (d) hexameric IgG2a-mouse antibodies. (e) Hexameric IgG-mouse antibodies, and (f) hexameric IgG1-rat antibodies. The white circle in (e) indicates the area where we can see many Fc-ring features of the hexamers.

We deposited these IgG antibodies on the mica substrate in the same manner as in a previous study at room temperature.^12^ Five minutes later, the substrate was rinsed five times with a phosphate buffer solution containing 50 mM magnesium chloride. We performed AFM imaging without drying the sample. Fig. 2a shows the IgG1-mouse-A hexamer formed by the Fc–Fc interaction. We observed donut-like structures consisting of six Fc regions at the center of the IgG hexamer. The hexamers form 2D crystals by the Fab–Fab interaction (Fig. 2a). Fig. 2b, a magnified image, shows that the X-shaped structure is formed by four comma-shaped Fab regions from the neighboring four antibodies. Even the subdomains in the Fab regions are clearly resolved as globular structures as indicated in the image. It was revealed from the AFM observation that the X-shaped structure does not have a fourfold symmetry but a twofold symmetry. We found from the AFM image that two Fab regions, indicated by the blue arrows, facing in a diagonal arrangement are directly interacting with each other to form a pair, and the other two Fab regions, indicated by the red arrows, interact with the side of a pair of the Fab regions.

Fig. 2c shows an AFM image of the IgG1-mouse-B molecules showing that they also form hexamers, which is reasonable because the hexamer formation is mediated by the Fc–Fc interaction and the Fc region is constant for the IgG1-mouse-A and IgG1-mouse-B. On the other hand, the IgG1-mouse-B hexamers did not form the 2D crystal (See Fig. S1 in ESI† for AFM image of IgG1-mouse-B hexamers prepared with the antibody solution with a higher concentration). This is probably because the Fab–Fab interaction of the IgG1-mouse-B was weaker than that of the IgG1-mouse-A. Since the only difference between the IgG1-mouse-A and IgG1-mouse-B molecules is the CDR, the Fab–Fab interaction that mediates the 2D crystallization is specific to the IgG1-mouse-A antibody. It is suggested that some specific amino residues in the CDR of the IgG1-mouse-A are interacting with the CDR or the Fab arms in the IgG1-mouse-A.

Fig. 2d shows the hexamers of the IgG2a antibodies (IgG2a-mouse). This is also reasonable because the IgG2a and the IgG1 are in the same isotype (IgG) and they share similar heavy chains.^23^ Namely, the Fc region in the heavy chains in the IgG2a-mouse is very similar to that of the IgG1-mouse-A and IgG1-mouse-B. It is suggested that the IgG hexamerization is a common phenomenon for IgG molecules of all the subclasses from the mouse. Indeed, we found that the polyclonal antibodies from the mouse (IgG-mouse) also form hexamers as shown in Fig. 2e. We can see some Fc rings in the white circle overlaid in the figure. Since the IgG2a and other IgG antibodies having similar Fc regions are known to have a strong affinity to the complement, except for IgG1,^21^ the hexamer mediated by the Fc–Fc interactions should be related to the complement binding. It is suggested that the IgG antibodies share the same amino sequences in the Fc domains that are responsible for the Fc–Fc interactions, but they are different sequences that are responsible for the binding to C1q, which are missing in the IgG1. Finally, we also studied the IgG antibody from the rat (IgG1-rat) as shown in Fig. 2f. In this figure, we can identify the Fc-ring consisting of six Fc regions, which means that they form the IgG hexamer. Although the IgG1 antibodies from the rat do not bind to the complement like the IgG1 from mouse,^24^ they share similar Fc regions with the other antibodies of different subclasses from the rat. Based on these results, we suggest that hexamerization is common for all the IgG antibodies from the mouse and rat, and they are mediated by the Fc–Fc interactions that are common for the similar Fc domains, while the IgG1 antibodies do not bind and activate the complement.

In the following section, we discuss the immunoactivity of the self-assembled hexamers of the antibodies. The antigen–antibody binding is described as
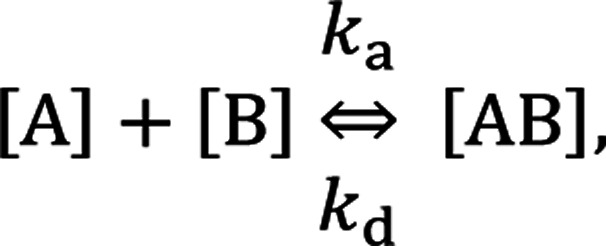
where [A], [B], and [AB] are the concentration of antigen, antibody and antigen–antibody complex, respectively, and *k*_a_ and *k*_d_ present the association and dissociation rate constants, respectively. The rate of formation of the complex is given by
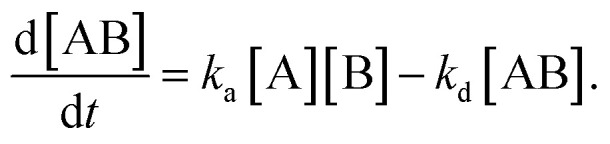


By defining [B] as [B] = *B*_0_ − [AB] where *B*_0_ is [B] at *t* = 0, and [A] as the constant *C*, d[AB]/d*t* can be rewritten as
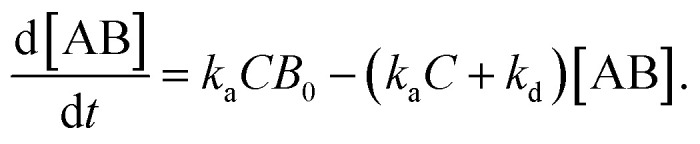


Since [AB] is proportional to the apparent coverage of the complex that can be measured by AFM, we can write the apparent coverage as *R* = *c*_AFM_[AB], where *c*_AFM_ is a constant. Now the rate of the apparent coverage of the antigen–antibody complex, d*R*/d*t*, is rewritten as
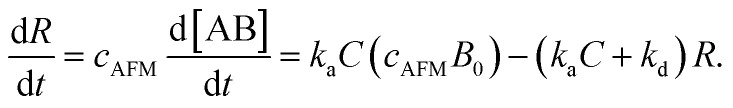


In the case when all antibodies bind antigenic molecules, [AB] becomes its maximum, [AB]_max_, which should be the same as the number of the antibodies before the interaction, namely *B*_0_. Therefore, the maximum apparent coverage of antigen–antibody complexes, *R*_max_ is given by *c*_AFM_[AB]_max_ = *c*_AFM_*B*_0_. Finally, we obtain the following equation.^25,26^
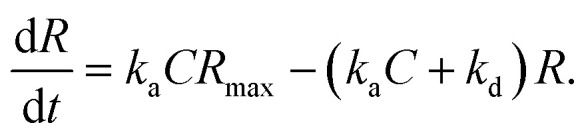


Since we didn't observe any desorption of the antigenic molecules on the 2D IgG crystal during AFM scans, we assumed the dissociation rate constant to be zero. This means the antigen molecules were strongly bound to the IgG antibodies in the crystal and the association was considered to be irreversible. Such an extremely stable binding have also been reported on early surface-based studies,^25,27^ in which the receptor molecules were directly immobilized on the solid surface. Therefore it could be related to the reduced flexibility of the Fab arms, which will be discussed later. Another possible reason for the slow dissociation is because the dissociated antigens immediately reassociated with the IgG antibodies before diffusing away from the surface since the mica surface was densely covered by the IgG antibodies with a high density.^25^

Although we cannot estimate the dissociation rate constant by the AFM measurement in this study, the association rate constant can be evaluated by plotting d*R*/d*t vs. R* if *R*_max_ is known. In the following, we demonstrate the estimation of the association rate constant by measuring d*R*/d*t* and *R*_max_ by the AFM. Since the IgG1-mouse-A was the only antibody whose hexamers form the 2D crystal, we used the IgG1-mouse-A molecules in the following experiments.

**Fig. 3:**
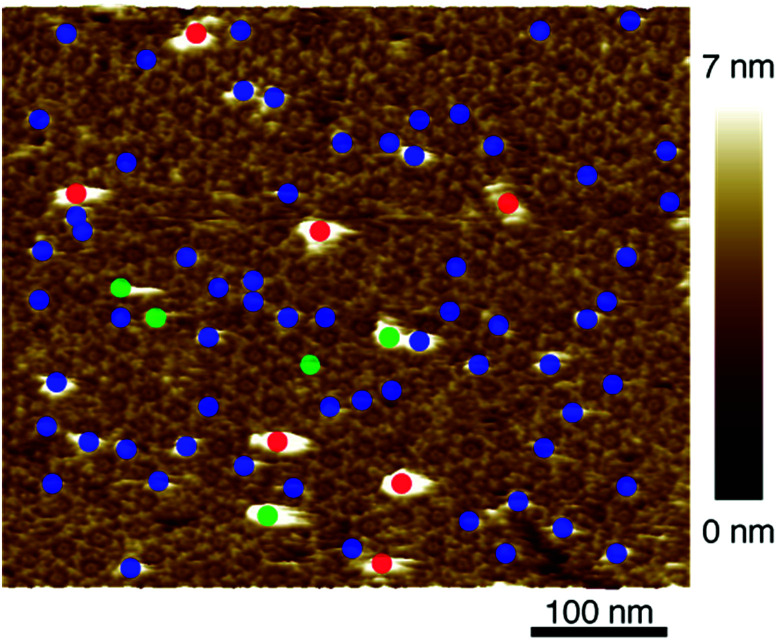
AFM image of 2D IgG crystal with antigenic molecules. The circles indicate the antigenic molecules binding to the Fab regions (blue), to the Fc regions (green), and to the other sites.

After confirmation of formation of 2D IgG crystals of IgG1-mouse-A, a 5 μl droplet of the antigen solution with a concentration of 0.08 μM was dropped onto the 2D IgG crystals. First, we investigated the adsorption sites of the antigenic molecules on the IgG antibodies. The adsorbed antigens were classified into the following three categories: the antigens binding to the Fab regions of the antibodies (blue), those binding to the Fc regions (green) and the remaining ones (red) (Fig. 3). We performed the same analysis for four images including Fig. 3 (see Table S1 and ESI Fig. 2†) and we found that most antigenic molecules adsorbed onto the Fab region. The average percentage of the antigen adsorption to the Fab region was 86%. This result reflects the specificity of the antibody–antigen binding.

**Fig. 4:**
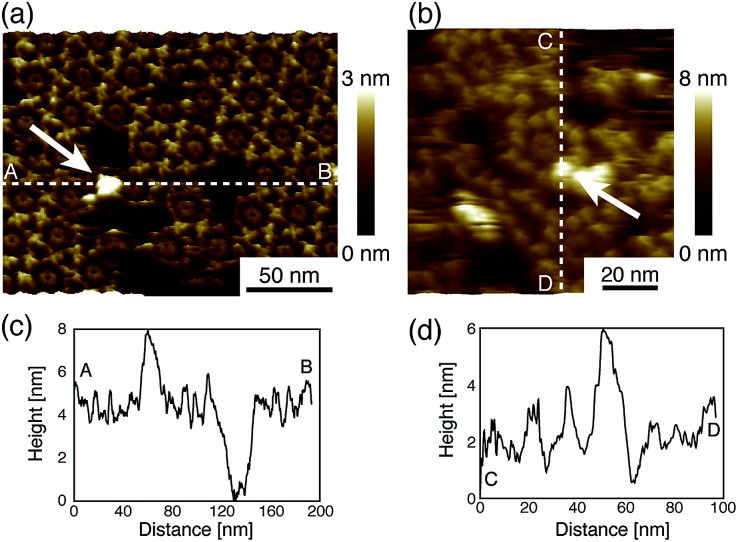
AFM images of antibody–antigen binding to (a) 2D IgG crystals (b) IgG hexamers. The white arrows indicate the antigenic molecule binding to the Fab region.

Next, we investigated the binding site of the antigen molecules to the Fab region by high-resolution AFM imaging. We used the IgG1-mouse-A and IgG1-mouse-B antibodies in this experiment. Fig. 4a and b show the antigen molecules adsorbed on the Fab regions after dropping an antigen solution with a concentration of 0.08 μM. The cross-sectional profile along the dashed lines A–B and C–D are shown in Fig. 4c and d. The white arrows in both images indicate the antigenic molecules. The IgG antibodies forming 2D crystals bind the antigenic molecule at the X-shaped structure that consists of four Fab regions. The heights of the antigenic molecules from the Fab regions were about 2 nm that is consistent with the histogram in Fig. 6b. Based on these experiments, it is confirmed that antigenic molecules are bound to Fab regions of antibodies, which assures that the apparent coverage, *R*, is a measure of the density of the complex, [AB].

**Fig. 5:**
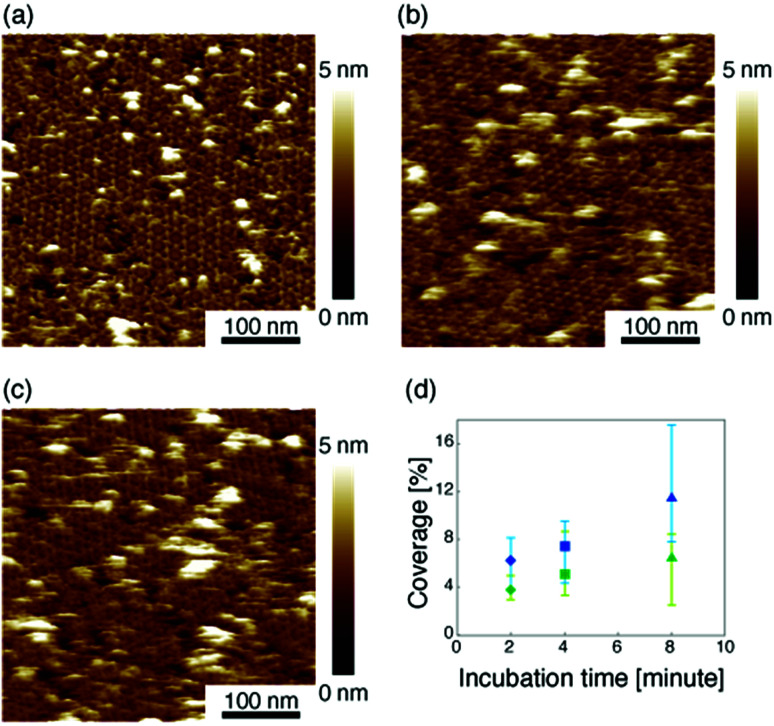
(a)–(c) AFM images of 2D IgG crystals taken at (a) 2 minutes, (b) 4 minutes, and (c) 8 minutes after dropping an antigen solution with a concentration of 3.0 μM. (d) Apparent coverage as a function of the incubation time. The antigen concentrations were 0.08 μM (green) and 0.3 μM (blue).

**Fig. 6:**
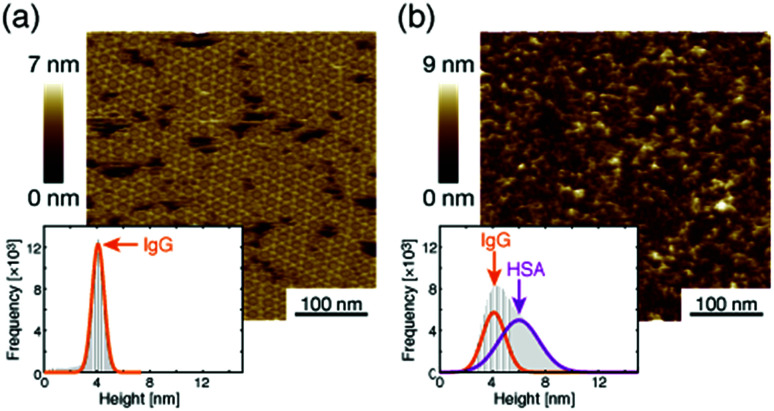
AFM image of 2D IgG crystals (a) before and (b) after dropping the excess antigenic molecules.

Then we measured the apparent coverage of the antigen molecules as a function of the incubation time (d*R*/d*t*) and the maximum apparent coverage (*R*_max_). We confirmed the 2D IgG crystal formation as shown in Fig. 2a by dropping a 5 μl droplet of antigenic molecules onto the 2D IgG crystals. The substrate was rinsed five times with the same imaging solution after five minutes. We performed AFM imaging without drying the sample. Fig. 5a–c show the AFM images at 2, 4, and 8 minutes after the addition of the antigenic molecules with a concentration of 0.3 μM. In this experiment, the antigen concentrations were set at 0.08 μM and 0.3 μM, and we found that the apparent coverage increased faster at the higher antigen concentration (Fig. 5d). Note that we confirmed that the adsorption of the antigens were not diffusion-limited since the rate of formation of the complex did not follow the formula of the diffusion-limited association,^25^ which is given by
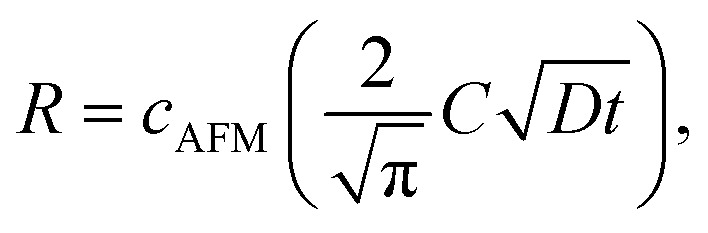
where *D* is a diffusion constant of the antigenic molecule in the solution. This means that the concentration was sufficiently high for the estimation of the association rate constant. We then dropped excess antigenic molecules and measured the maximum apparent coverage (*R*_max_). Fig. 6a and b show the AFM images before and after the addition of the excess antigens by dropping an antigen solution with a concentration of 3.0 μM, respectively. Before adding the antigenic molecules, there is only one peak at the height of the 2D IgG crystals (4 nm) in the histogram. After adding the antigenic molecules, the topography drastically changed and there are two peaks at 4 nm and 6 nm in the histogram. The peak at 4 nm shows the height of the 2D IgG crystals and the peak at 6 nm corresponds to the sum of the height of the crystal and the antigenic molecule. By analyzing the histogram, we found that the apparent antigen coverage on the surface saturated at 45%. Taking the tip convolution effect into account, we estimated that the number of antigenic molecules per hexamer in the IgG 2D crystal as 3 (see ESI†). Considering that the number of Fab regions per hexamer (6 IgG molecules) is 12, the number of antigenic molecules per hexamer in the IgG 2D crystal is small by a factor of 4. This is probably because the antigen binding site in the IgG 2D crystal, X-shaped structure, is contributed by the four Fab arms from the two hexamers which are very close with each other, which causes a steric hindrance for binding of multiple antigenic molecules. Nevertheless, we consider that antigenic molecules bound to all the accessible Fab regions (One Fab region for each X-shaped structure) in the IgG 2D crystal on the mica substrate.

**Fig. 7:**
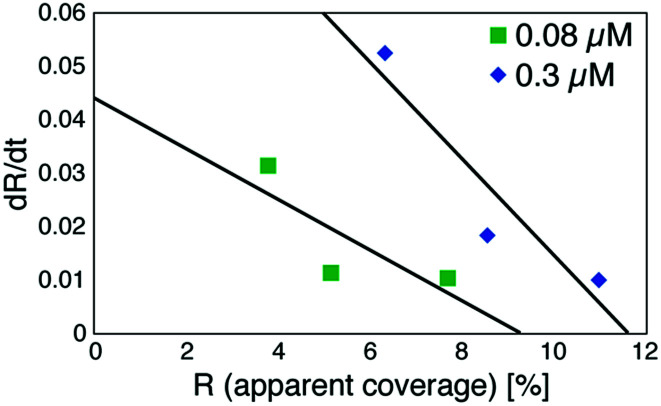
Plot of a time-differential of the apparent coverage of antigenic molecules bound to 2D IgG crystals (d*R*/d*t*) as a function of *R*.

Based on these experiments, by plotting d*R*/d*t* as a function of *R* as shown in Fig. 7 (see Table S2† for values), we evaluated the association rate constant at 0.08 μM and 0.3 μM to be 6 × 10^4^ M^−1^ s^−1^ and 3 × 10^4^ M^−1^ s^−1^, respectively. The reason why the estimated value was higher for a lower antigen concentration was probably because the apparent coverage was overestimated in the low coverage regime due to a finite tip radius of the AFM. The estimated values of the order of 10^4^ M^−1^ s^−1^ was about ten times lower than the reported association rate constants measured by surface plasmon resonance (SPR)^26,28^ and solution-based analytical methods such as isothermal titration calorimetry.^29^ We consider that the reduced flexibility of the Fab arms on the substrate, as well as the limited accessibility to the binding sites for the antigenic molecules as mentioned above, reduced the association rate constant. This is in contrast with that the association rate constant measured by SPR was almost same as those measured by the solution-based methods because of the use of the polymer layer such as dextran for immobilization of the antibodies on the sensor chip.^30,31^ In this study, we could not discuss the binding mechanisms such as conformation selection from the adsorption curves. However, we consider that, by tuning the immobilization conditions and thereby controlling the flexibility of the IgG antibodies on the solid surface following the methodology of the SPR, investigations of the antibody–antigen interactions by using AFM could give useful information not only on the immunoactivities of the antibodies on the solid surface but also on the specific interactions between various biomolecules as well as other surface-based and solution-based analytical methods. Finally, it should be noted here that another possible reason for the reduced association rate constant was the allosteric control of the IgG1 binding to the HSA by hexamer formation or the crystallization. While we only measured the HSA adsorption on the IgG1-mouse-A crystal in this study, we are planning to measure the HSA adsorption on isolated hexamers as well as isolated antibodies as a future study, that will allow us to discuss the effect of the self-assembly on the affinity constant.

## Conclusions

In summary, we investigated the generality and immunoactivity of the IgG self-assembled hexamers in an aqueous solution by AFM. We found that the IgG hexamerization was a relatively general phenomenon for the IgG antibodies from mouse and rat, but the 2D crystallization was a phenomenon specific to the antibody IgG1-mouse-A among the antibodies we have studied. The results suggested that the hexamer formation is related to the function of the IgG antibodies, especially the complement binding and activation. Although the IgGl antibodies from mouse that we mainly focused in this paper do not have a complement activation capability, unfortunately, we are planning to study if C1q bind to these IgG hexamers in the future.

We confirmed that the antigenic molecules were specifically bound on the Fab regions of the hexameric IgG molecules by a statistical analysis of the AFM images as well as by the high-resolution AFM imaging, and then estimated the association rate constant of the IgG antibodies on the mica substrate as about 10^4^ M^−1^ s^−1^. Although the estimated value was about one order of magnitude lower than those measured by the conventional method probably because of the reduced flexibility of the Fab arms, AFM measurements on the solid surface with an enhanced flexibility of the molecules would give useful information on the specific interactions between various biomolecules. While conventional analytical methods allow us to obtain averaged information, AFM imaging is one of the few promising methods that could provide us with local information in liquids such as molecular conformations and their flexibility, and their binding domains.

## Conflicts of interest

There are no conflicts to declare.

## Supplementary Material

RA-008-C8RA05423A-s001
