# Supplementary material for: Immunoactivity of self-assembled antibodies investigated by atomic force microscopy
Source: RSC Adv. 2018 Aug 20;8(51):29378–84. doi: 10.1039/c8ra05423a (PMC9084463; doi:10.1039/c8ra05423a)
Supplement: RA-008-C8RA05423A-s001 [file RA-008-C8RA05423A-s001.pdf]

Electronic Supplementary Material (ESI) for RSC Advances.
This journal is © The Royal Society of Chemistry 2018

Supporting Information

Immunoactivity of self-assembled antibodies investigated by atomic force microscopy

Hiroaki Kominami,[a] *Shinichiro Ido*,[a] *Hirokazu Kimiya*,[b] *Kei Kobayashi*,[a] *and Hirofumi Yamada*[*a]

[a]Department of Electronic Science and Engineering, Kyoto University, Katsura, Nishikyo, Kyoto, 615-8510, Japan

[b]*Advanced Research Division Device Research Laboratory, Panasonic Corporation 3-1-1 Yagumo-naka-machi, Moriguchi City, Osaka 570-8501, Japan*

Corresponds should be addressed to Hirofumi Yamada (h-yamada@kuee.kyoto-u.ac.jp).

Supporting methods

Calculation of bound antigenic molecules. We first considered a trajectory of a parabolic AFM tip with a radius of curvature R_a during a scan on a spherical sample with a radius of R_s.[2] The apparent width (W) of the spherical sample in the AFM image is given by $W = 4(R_a + R_s)\sqrt{R_s(R_a - R_s)}/R_a$. By assuming R_a as 7 nm from the nominal tip radius as obtained from the manufacturer, and R_s as 1 nm from the half of the apparent height of the HSA, we estimated W to be 11 nm. Since the apparent area of the antigenic molecule was about 100 nm^2, the number of the antigenic molecules in the 45% area in the scan area (500 × 500 nm) in Fig. 4b was 1125. On the other hand, the number of unit cells (680 nm^2, see Fig. 1b) in the scan area was 368. From these figures the number of the bound antigenic molecules per unit cell was determined as 3.

Supporting reference

1. C. Bustamante, J. Vesenka, C. L. Tang, W. Rees, M. Guthold, R. Keller, *Biochemistry* 1992, **31**, 22–26.

Supporting figures and table

Table S1. Number of antigenic molecules binding to 2D IgG crystal (percentage / counts).

Fab region	Fc region	Other regions
84% / 64	7% / 5	9% / 7
82% / 50	7% / 4	11% / 7
87% / 60	6% / 4	7% / 5
92% / 60	5% / 3	3% / 2

Table S2. *R* values as a function of d*R*/d*t*.

	0.08 μM			0.3 μM		
d*R*/d*t*	0.0315	0.0114	0.0105	0.0527	0.0185	0.0101
R	3.78	5.15	7.67	6.32	8.54	10.96

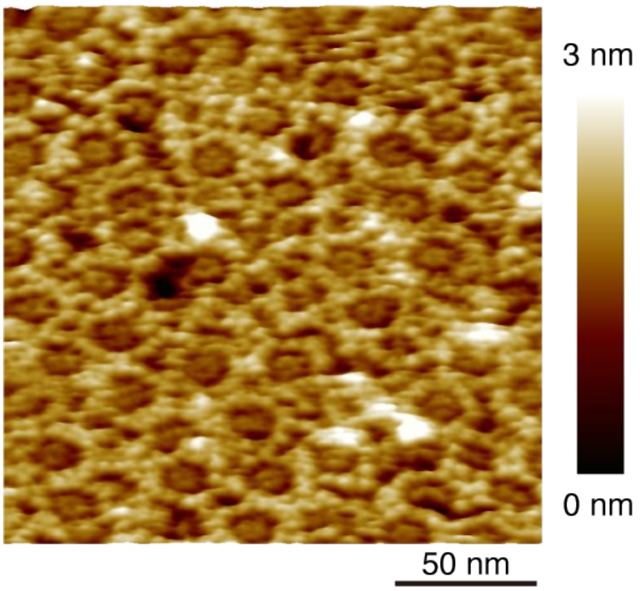

Figure S1. FM-AFM image of hexameric IgG1-mouse-B antibodies obtained after dropping the antibody solution of a concentration of 2.0 μM. The Fc-ring structures of the hexamers were clearly observed, but the IgG hexamers were not hexagonally arranged.

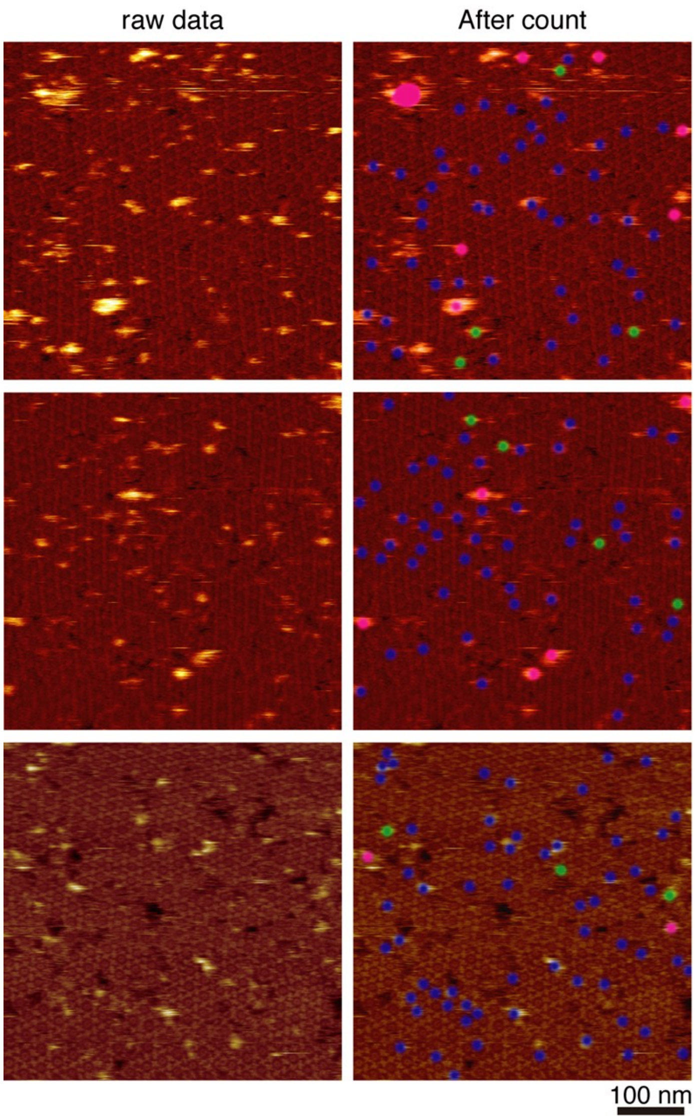

Figure S2. FM-AFM images of 2D IgG crystal with antigenic molecules. Original images and the images with counting markers are shown in left and right columns, respectively. Blue, green, and red markers indicate the antigens on the Fab region, Fc regions, and other regions, respectively.
